# Raw data of the effects of Chlorogenic acid in 3-Nitropropionic acid induced toxicity and genotoxicity

**DOI:** 10.1016/j.dib.2017.07.004

**Published:** 2017-07-12

**Authors:** Alarcón-Herrera Norberto, Flores-Maya Saúl, Bellido Belén, M. García-Bores Ana, Mendoza Ernesto, Ávila-Acevedo Guillermo, Hernández-Echeagaray Elizabeth

**Affiliations:** a Laboratorio de Recursos Naturales, UBIPRO, FES-Iztacala, Universidad, Nacional Autónoma de México, México; b Laboratorio de Neurofisiología del desarrollo y la neurodegeneración UBIMED, FES-Iztacala, Universidad, Nacional Autónoma de México, México; c Laboratorio de Fitoquímica, UBIPRO, FES-Iztacala, Universidad, Nacional Autónoma de México, México

**Keywords:** 3-NP, CGA, Toxicity, Genotoxicity, Data

## Abstract

The raw data showed in this article comes from the published research article entitled “Protective effects of Chlorogenic acid in 3-Nitropropionic acid induced toxicity and genotoxicity” Food Chem Toxicol. 2017 May 3. pii: S0278-6915(17)30226-0. DOI:10.1016/j.fct.2017.04.048. [1]. Data illustrates antitoxic and antigenotoxic effects of Chlorogenic acid (CGA) on toxicity and genotoxicity produced by the *in vivo* treatment with mitochondria toxin 3-Nitropropionic acid (3-NP) in mice. Toxicity and genotoxicity was evaluated in erythrocytes of peripheral blood through the micronuclei assay. Data was share at the Elsevier repository under the reference number FCT9033.

**Specifications Table**

**Table:** 

Subject area	*Biology*
More specific subject area	*Toxicology*
Type of data	*Tables*
How data was acquired	*Data from erythrocytes from peripheral blood, stained with H&E were classified in Normochromatic, Polychromatic or Polychromatic with micronuclei cells. 1000 cells per each condition were counted with the aid of by the aid of an optic microscope (Leica, Microsystems AG)*.
Data format	*Raw data*
Experimental factors	*In order to determine toxicity and genotoxicity of 3-NP, as well as protective effects of CGA, 6 experimental groups were evaluated; Negative control, Control (PB), 3-NP, CGA, 3-NP + CA, P/CA, 3-NP + CA and P/CA, 3-NP*
	*Each treatment lasted for 5 days except those where 5 days of pretreatment was present*.
Experimental features	*To evaluate toxic and genotoxic effect of 3-NP and the antitoxic and antigenotoxic effects of CGA in erythrocytes*.
Data source location	*México City, México*
Data accessibility	*Data are available in this article and were place also in a public repository provided by Elsevier submission system, under reference number FCT9033*

**Value of the data**

• Data displays Normochromatic, Polychromatic or Polychromatic cells with micronuclei in the different experimental conditions and can be used by other research groups.
• Toxicity and Genotoxicity were measured in peripheral blood by using the micronuclei assay.
• Τhese data are important because few studies have carried out to evaluate toxicity and genotoxicity of 3-NP which can be found in plants like sugar cane that are eaten by cattle and humans.
• Data about CGA protective effects are important because CGA is found in a variety of food products which can help to protect the health of living organisms.

## 1 Data

Raw data presented in this paper gives information about protective role of CGA on toxic and genotoxic effects of 3-NP in erythrocytes from peripheral blood. Data are shown in Table 1, Table 2, Table 3, Table 4, Table 5, Table 6, Table 7 which illustrate the effect in each evaluated time.

**Table 1:** Data obtained at 24 h measurements. Normochromatic erythrocyte cells (CN), Polychromatic erythrocyte cells (PC), Polychromatic cells with micronuclei (PMNC), Toxicity (TOX), Genotoxicity (Gen), 3-Nitropropionic acid (3-NP), Chlorogenic acid (CGA), Pretreatment of Chlorogenic acid (P/CGA).

**24 h**					
**Treatment**	**Negative Control**				
Animal	CN	PC	PMNC	%TOX	%GEN
1	808	192	5	19.2	0.5
2	803	197	5	19.7	0.5
3	784	216	4	21.6	0.4
4	798	202	2	20.2	0.2
5	813	187	2	18.5	0.2
**Treatment**	**Ifosfamide**				
Animal	CN	PC	PMNC	%TOX	%GEN
1	860	149	21	14.9	2.1
2	875	125	22	12.5	2.2
3	801	199	25	19.9	2.5
4	855	145	20	14.5	2
5	831	169	13	16.9	1.3
**Treatment**	**Buffer**				
Animal	CN	PC	PMNC	%TOX	%GEN
1	811	189	3	18.9	0.3
2	787	213	5	21.3	0.5
3	771	229	2	22.9	0.2
4	814	186	4	18.6	0.4
5	805	195	3	19.5	0.3
**Treatment**	**3-NP**				
Animal	CN	PC	PMNC	%TOX	%GEN
1	878	122	20	12.2	2
2	889	111	19	11.1	1.9
3	895	105	15	10.5	1.5
4	917	83	15	8.3	1.5
5	933	97	13	9.7	1.3

**Treatment**	**CGA**				
Animal	CN	PC	PMNC	%TOX	%GEN
1	835	165	3	16.5	0.3
2	828	172	5	17.2	0.5
3	817	183	4	18.3	0.4
4	810	190	3	19	0.3
5	812	188	2	18.8	0.2

**Treatment**	**3NP + CGA**				
Animal	CN	PC	PMNC	%TOX	%GEN
1	843	157	10	15.7	1
2	840	160	10	16.0	1
3	835	165	13	16.5	1.3
4	810	190	11	19.0	1.1
5	812	188	12	18.8	1.2

**Treatment**	**P/CGA, 3NP + CGA**				
Animal	CN	PC	PMNC	%TOX	%GEN
1	797	203	6	20.3	0.6
2	813	187	8	18.7	0.8
3	801	199	3	19.9	0.3
4	796	204	3	20.4	0.3
5	807	193	5	19.3	0.5
1	830	170	11	17	1.1
2	839	161	9	16.1	0.9
3	848	152	10	15.2	1
4	885	115	8	11.5	0.8
5	855	145	8	14.5	0.8

**Table 2:** Data obtained at 48 h measurements. Normochromatic erythrocyte cells (CN), Polychromatic erythrocyte cells (PC), Polychromatic cells with micronuclei (PMNC), Toxicity (TOX), Genotoxicity (Gen), 3-Nitropropionic acid (3-NP), Chlorogenic acid (CGA), Pretreatment of Chlorogenic acid (P/CGA).

**48** **h**							
**Treatment**	**Negative control**						
Animal	CN	PC	PMC	%TOX		%GEN	
1	789	211	5	21.1		0.5	
2	783	217	3	21.7		0.3	
3	810	190	6	19		0.6	
4	801	194	5	19.4		0.5	
5	810	190	1	19		0.1	
**Treatment**	**Ifosfamide**						
Animal	CN	PC	PMNC	%TOX			%GEN
1	857	143	15	14.3			1.5
2	862	148	17	14.8			1.7
3	877	123	15	12.3			1.5
4	859	141	18	14.2			1.8
5	865	135	18	13.5			1.8
**Treatment**	**Buffer**						
Animal	CN	PC	PMNC	%TOX			%GEN
1	817	183	5	18.3			0.5
2	807	193	6	19.3			0.6
3	760	240	5	24			0.5
4	820	180	5	18			0.5
5	790	210	5	21			0.5
**Treatment**	**3-NP**						
Animal	CN	PC	PMNC	%TOX			%GEN
1	870	130	11	13			1.1
2	891	109	10	10.9			1
3	816	184	13	18.4			1.3
4	850	150	10	15			1
5	877	123	10	12.3			1
**Treatment**	**CGA**						
Animal	CN	PC	PMNC	%TOX			%GEN
1	793	207	4	20.7			0.4
2	806	194	7	19.4			0.7
3	807	193	5	19.3			0.5
4	800	200	6	20			0.6
5	810	190	5	19			0.5
**Treatment**	**3-NP + CGA**						
Animal	CN	PC	PMNC	%TOX			%GEN
1	847	153	10	15.3			1
2	855	165	11	16.5			1.1
3	833	167	10	16.7			1
4	832	168	11	16.8			1.1
5	827	183	12	18.3			1.2
**Treatment**	**P/CGA, 3-NP + CGA**						
Animal	CN	PC	PMNC	%TOX			%GEN
1	797	203	2	20.3			0.2
2	830	170	4	17			0.4
3	794	206	2	20.6			0.2
4	788	212	6	21.2			0.6
5	799	201	5	20.1			0.5
**Treatment**	**P/CGA, 3-NP**						
Animal	CN	PC	PMNC	%TOX	%GEN		
1	857	163	7	16.3	0.7		
2	818	182	8	18.2	0.8		
3	870	130	10	13	1		
4	876	128	2	12.8	0.2		
5	820	180	6	18	0.6

**Table 3:** Data obtained at 72 h measurements. Normochromatic erythrocyte cells (CN), Polychromatic erythrocyte cells (PC), Polychromatic cells with micronuclei (PMNC), Toxicity (TOX), Genotoxicity (Gen), 3-Nitropropionic acid (3-NP), Chlorogenic acid (CGA), Pretreatment of Chlorogenic acid (P/CGA).

**72** **h**					
**Treatment**	**Negative Control**				
Animal	CN	PC	PMNC	%TOX	%GEN
1	794	205	3	20.5	0.3
2	799	201	3	20.1	0.3
3	799	201	3	20.1	0.3
4	791	209	2	20.9	0.2
5	795	205	3	20.5	0.3
**Treatment**	**Ifosfamide**				
Animal	CN	PC	PMNC	%TOX	%GEN
1	832	168	25	16.8	2.5
2	838	162	26	16.2	2.6
3	751	249	25	24.9	2.5
4	885	175	24	17.5	2.5
5	783	217	17	21.7	1.7
**Treatment**	**Buffer**				
Animal	CN	PC	PMNC	%TOX	%GEN
1	803	197	5	19.7	0.5
2	801	199	4	19.9	0.4
3	756	244	5	24.4	0.5
4	826	174	4	17.4	0.4
5	800	200	5	20	0.5
**Treatment**	**3-NP**				
Animal	CN	PC	PMNC	%TOX	%GEN
1	879	121	20	12.1	2
2	862	138	17	13.8	1.7
3	860	140	17	14	1.7
4	838	162	19	16.2	1.9
5	855	145	17	14.5	1.7

**Treatment**	**CGA**				
Animal	CN	PC	PMNC	%TOX	%GEN
1	799	201	4	20.1	0.4
2	827	173	5	17.3	0.5
3	785	215	4	21.5	0.4
4	808	192	8	19.2	0.8
5	800	200	6	20	0.6

**Treatment**	**3-NP + CGA**				
Animal	CN	PC	PMNC	%TOX	%GEN
1	820	180	9	18	0.9
2	821	179	9	17.9	0.9
3	816	184	12	18.4	1.2
4	810	190	11	19	1.1
5	815	185	10	8.5	1

**Treatment**	**P/CGA, 3-NP + CGA**				
Animal	CN	PC	PMNC	%TOX	%GEN
1	790	210	5	21	0.5
2	795	205	3	20.5	0.3
3	796	204	2	20.4	0.2
4	789	211	3	21.1	0.3
5	797	203	4	20.3	0.4
**Treatment**	**P/CGA, 3-NP**				
Animal	CN	PC	PMNC	%TOX	%GEN
1	846	154	7	15.4	0.7
2	832	168	7	16.8	0.7
3	868	132	10	13.2	1
4	857	143	5	14.3	0.5
5	849	151	9	15.1	0.9

**Table 4:** Data obtained at 96 h measurements. Normochromatic erythrocyte cells (CN), Polychromatic erythrocyte cells (PC), Polychromatic cells with micronuclei (PMNC), Toxicity (TOX), Genotoxicity (Gen), 3-Nitropropionic acid (3-NP), Chlorogenic acid (CGA), Pretreatment of Chlorogenic acid (P/CGA).

**96** **h**					
**Treatment**	**Negative Control**				
Animal	CN	PC	PMNC	%TOX	%GEN
1	787	213	5	21.3	0.5
2	813	187	3	18.7	0.3
3	817	183	1	18.3	0.1
4	793	207	6	20.7	0.6
5	799	201	5	20.1	0.5
**Treatment**	**Buffer**				
Animal	CN	PC	PMNC	%TOX	%GEN
1	804	196	6	19.6	0.6
2	792	208	5	20.8	0.5
3	788	212	3	21.2	0.3
4	810	190	4	19	0.4
5	803	197	5	19.7	0.5
**Treatment**	**3-NP**				
Animal	CN	PC	PMNC	%TOX	%GEN
1	855	145	12	14.5	1.2
2	880	120	22	12	2.2
3	848	152	11	15.2	1.1
4	872	128	13	12.8	1.3
5	847	153	13	15.3	1.3

**Treatment**	**CGA**				
Animal	CN	PC	PMNC	%TOX	%GEN
1	803	197	5	19.7	0.5
2	801	199	4	19.9	0.4
3	810	190	6	19	0.6
4	784	216	6	21.6	0.6
5	797	203	5	20.3	0.5

**Treatment**	**P/CGA, 3-NP + CGA**				
Animal	CN	PC	PMNC	%TOX	%GEN
1	796	204	3	20.4	0.3
2	799	201	4	20.1	0.4
3	801	199	4	19.9	0.4
4	789	211	4	21.1	0.4
5	795	205	5	20.5	0.5
**Treatment**	**P/CGA, 3-NP**				
Animal	CN	PC	PMNC	%TOX	%GEN
1	825	175	7	17.5	0.7
2	834	166	5	16.6	0.5
3	796	204	6	20.4	0.6
4	820	180	7	18	0.7
5	853	147	10	14.7	1

**Table 5:** Data obtained at 120 h measurements. Normochromatic erythrocyte cells (CN), Polychromatic erythrocyte cells (PC), Polychromatic cells with micronuclei (PMNC), Toxicity (TOX), Genotoxicity (Gen), 3-Nitropropionic acid (3-NP), Chlorogenic acid (CGA), Pretreatment of Chlorogenic acid (P/CGA).

**120** **h**					
**Treatment**	**Negative Control**				
Animal	CN	PC	PMNC	%TOX	%GEN
1	803	197	3	19.7	0.3
2	769	231	5	23.1	0.5
3	796	204	2	20.4	0.2
4	803	193	3	19.3	0.3
5	805	195	3	19.5	0.3

**Treatment**	**Buffer**				
Animal	CN	PC	PMNC	%TOX	%GEN
1	805	195	3	19.5	0.3
2	816	184	4	18.4	0.4
3	766	234	5	23.4	0.5
4	808	192	3	19.2	0.3
5	789	211	4	21.1	0.4
**Treatment**	**3-NP**				
Animal	CN	PC	PMNC	%TOX	%GEN
1	888	112	10	11	1
2	873	127	16	12.7	1.6
3	868	132	17	13.2	1.7
4	863	137	16	13.7	1.6
5	871	129	15	12.9	1.5
		
**Treatment CGA**					
Animal	CN	PC	PMNC	%TOX	%GEN
1	820	180	4	18	0.4
2	793	207	5	20.7	0.5
3	779	221	4	22.1	0.4
4	800	200	6	20	0.6
5	808	192	10	19.2	1

**Treatment**	**3-NP + CGA**				
Animal	CN	PC	PMNC	%TOX	%GEN
1	824	176	17	17.6	1.7
2	809	191	14	19.1	1.4
3	817	183	15	18.3	1.5
4	821	179	16	17.9	1.6
5	817	183	15	18.3	1.5

**Treatment**	**P/CGA, 3-NP + CGA**				
Animal	CN	PC	PMNC	%TOX	%GEN
1	787	213	3	21.3	0.3
2	819	181	3	18.1	0.3
3	785	215	3	21.5	0.3
4	800	200	4	20	0.4
5	771	229	4	22.9	0.4
**Treatment**	**P/CGA, 3-NP**				
Animal	CN	PC	PMNC	%TOX	%GEN
1	816	184	9	18.4	0.9
2	824	176	8	17.6	0.8
3	783	217	7	21.7	0.8
4	829	171	7	17.1	0.7
5	858	142	12	14.2	1.2

**Table 6:** Data obtained at 144 h measurements. Normochromatic erythrocyte cells (CN), Polychromatic erythrocyte cells (PC), Polychromatic cells with micronuclei (PMNC), Toxicity (TOX), Genotoxicity (Gen), 3-Nitropropionic acid (3-NP), Chlorogenic acid (CGA), Pretreatment of Chlorogenic acid (P/CGA).

**144** **h** (6 days)					
**Treatment**	**Negative Control**				
Animal	CN	PC	PMNC	%TOX	%GEN
1	802	198	2	19.8	0.2
2	793	207	3	20.7	0.3
3	799	201	0	20.1	0
4	802	198	3	19.8	0.3
5	816	184	2	18.4	0.2

**Treatment**	**Buffer**				
Animal	CN	PC	PMNC	%TOX	%GEN
1	806	194	2	19.4	0.2
2	809	191	4	19.2	0.4
3	773	227	5	22.7	0.5
4	819	181	4	18.1	0.4
5	807	193	3	19.3	0.3
**Treatment**	**3-NP**				
Animal	CN	PC	PMNC	%TOX	%GEN
1	851	149	19	14.9	1.9
2	877	123	16	12.3	1.6
3	837	163	19	16.3	1.9
4	840	160	17	16	1.7
5	845	155	17	15.5	1.7
**Treatment**	**3-NP+ CGA**				
Animal	CN	PC	PMNC	%TOX	%GEN
1	833	167	11	6.7	1.1
2	814	186	12	18.6	1.2
3	821	179	15	17.9	1.5
4	836	165	13	16.5	1.3
5	820	180	15	18	1.5
**Treatment**	**P/CGA, 3-NP + CGA**				
Animal	CN	PC	PMNC	%TOX	%GEN
1	770	230	2	23	0.3
2	795	205	3	20.5	0.3
3	767	233	3	23.3	0.3
4	766	234	3	23.4	0.3
5	753	247	4	24.7	0.4
**Treatment**	**P/CGA, 3-NP**				
Animal	CN	PC	PMNC	%TOX	%GEN
1	830	170	5	17	0.5
2	817	183	6	18.3	0.6
3	813	187	12	18.7	1.2
4	825	175	12	17.5	1.2
5	827	173	7	17.3	0.7

**Table 7:** Global Toxicity and Genotoxicity obtained by averaging data condition for every time evaluated are display in this table. Normochromatic erythrocyte cells (CN), Polychromatic erythrocyte cells (PC), Polychromatic cells with micronuclei (PMNC), Toxicity (TOX), Genotoxicity (Gen), 3-Nitropropionic acid (3-NP), Chlorogenic acid (CGA), Pretreatment of Chlorogenic acid (P/CGA).

**Global Toxicity**								
Time	Negative Control	Ifosfamide	Buffer	3-NP	CGA	3-NP + CGA	P/CGA, 3-NP + CGA	P/CGA, 3-NP
24 h	19.84	15.74	20.24	10.36	17.96	17.2	19.72	14.86
48 h	20.04	13.82	20.12	13.92	19.68	16.72	19.84	15.66
72 h	20.42	19.42	20.28	14.12	19.62	16.36	20.66	14.96
96 h	19.82		20.06	13.96	20.1	17.86	20.4	17.44
120 h	20.4		20.32	12.7	20	18.24	20.76	17.8

**Global Genotoxicity**								
Time	Negative Control	Ifosfamide	Buffer	3-NP	CGA	3-NP + CGA	P/CGA, 3-NP+ CGA	P/CGA, 3-NP
24 h	0.36	2.02	0.34	1.64	0.34	1.12	0.5	0.92
48 h	0.4	1.66	0.52	1.08	0.54	1.08	0.38	0.66
72 h	0.28	2.36	0.46	1.8	0.54	1.02	0.34	0.76
96 h	0.4		0.46	1.42	0.52	1.66	0.4	0.7
120 h	0.32		0.38	1.48	0.58	1.54	0.34	0.88

## 2 Experimental design, materials and methods

To evaluate toxic effect of 3-NP and the antitoxic effect of Chlorogenic acid, mice were randomly assigned to one of the following experimental groups: negative control, ifosfamide (positive control), buffer, 3-nitropropionic acid (3-NP), Chlorogenic acid (CGA), 3-NP + Chlorogenic acid without pretreatment (3-NP + CA, W/P), 3-NP + Chlorogenic acid together with pretreatment of CA per 5 days (P/CA, 3-NP + CA), pretreatment with CA for 5 days and later treatment of 3-NP alone, (P/CA, 3-NP).

All groups were treated for 5 days with i.p. doses of 3-NP (15 mg/kg), CGA (100 mg/kg).

Extraction and Characterization of CGA: Aerial parts of *B. scordioides* (300 g) were dried, ground and extracted with hexane and methanol in succession. The methanolic portion was evaporated under reduced pressure at 55 °C to obtain a syrup residue (30 g). CGA was isolated from methanolic extract by open column chromatography using SiO2 [2].

Micronuclei Assay: Samples of peripheral blood were obtained from the mice caudal vein at 24 h, 48 h, 72 h, 96 h, 120 h and 144 h after starting each treatment in each experimental group. A drop of blood was placed on a glass slide (3 slides per mice) and fixed with methanol for further H&E staining for 10 min.

Polychromatic and normochromatic cells of peripheral blood were counted with the aid of an optic microscope (Leica, Microsystems AG). Celle were classified in normochromatic (CN), polychromatic or (PC), polychromatic with micronuclei cells (MNC) in 1000 observed cells per each experimental condition in every evaluated time.

Genotoxicity index (%) and toxicity index (%) was calculated through the Hayashi and cols., method [3].

## Funding sources

This work was supported by CONACyT Grant no. 180660 to EHE.
